# Correlation between microRNA-143 in peripheral blood mononuclear cells and disease severity in patients with psoriasis vulgaris

**DOI:** 10.18632/oncotarget.17260

**Published:** 2017-04-20

**Authors:** Yi-Zhi Zheng, Chun-Feng Chen, Li-Ying Jia, Tu-Gen Yu, Jie Sun, Xiao-Yong Wang

**Affiliations:** ^1^ Department of Dermatology, The First Affiliated Hospital of Zhejiang Chinese Medical University, Hangzhou 310006, P.R. China; ^2^ Department of Mental Health, The First Affiliated Hospital of Zhejiang Chinese Medical University, Hangzhou 310006, P.R. China; ^3^ Department of Dermatology, Hangzhou Traditional Chinese Medical Hospital, Hangzhou 310007, P.R. China

**Keywords:** disease severity, microRNA-143, psoriasis vulgaris, peripheral blood mononuclear cell

## Abstract

This study aims to explore the correlation between microRNA-143 (miR-143) expression in peripheral blood mononuclear cells (PBMCs) and disease severity in patients with psoriasis vulgaris. From March 2014 to November 2015, 194 patients with psoriasis vulgaris (102 patients in progressive stage and 92 patients in stable stage) were selected as the case group and 175 healthy people as a control group were enrolled in this study. ELISA was used to detect the levels of IL-17 and VEGF in serum. The qRT-PCR assay was performed to detect the relative expression of miR-143 in PBMCs. Disease severity in psoriasis vulgaris patients was graded with Psoriasis Lesions Area and Severity Index (PASI). The value of miR-143 expression in PBMCs for the diagnosis of psoriasis vulgaris was evaluated using receiver operating characteristic (ROC) curve. The correlation between miR-143 expression in PBMCs and PASI scores was measured using Spearman rank correlation analysis. Compared with the control group, serum levels of IL-17 and VEGF were higher and miR-143 expression in PBMCs was lower in the case group. Furthermore, miR-143 expression in PBMCs was lower in patients in progressive stage than that in patients with stable stage. The relative expression of miR-143 in PBMCs was negatively correlated with PASI scores of patients with psoriasis vulgaris. ROC curve showed that miR-143 was a reliable and accurate biomarker of psoriasis vulgaris. Our findings suggest that miR-143 expression in PBMCs is negatively correlated the disease severity in psoriasis vulgaris.

## INTRODUCTION

Psoriasis, is a common inflammatory dermatosis with strongly hereditary and obvious regional and ethnic differences in its incidence rate [1]. Psoriasis onsets mainly at adolescence to young adulthood, and the incidence of psoriasis is increasing year by year [2, 3]. There are four types of psoriasis: psoriasis vulgaris, pustule psoriasis, erythrodermic psoriasis and arthropathic psoriasis [4]. Psoriasis vulgaris is the most common type, and is easy to diagnose but difficult to cure on account of its high recurrence rate [5]. The pathogenesis of this disease is not widely understood, but several factors are thought to be involved, including emotion, season, diet, alcohol drinking and smoking [6]. Physiotherapy, medication and Chinese medicinal therapy have been generally applied to cure the psoriasis, but each therapy has its own defect [7]. The latest study has demonstrated that microRNA (miR) is of high possibility to play a potential role in the gene regulation of psoriasis [8].

MiR is a non-coding small RNA, which has been evolutionarily conserved. It is coded by genomes of high eukaryotes and then regulates gene expression by translation [9]. An increasing number of studies have shown that specific miRs are linked with the pathology of psoriasis, such as miR181-b, miR-21 and miR-424 [10–12]. MiR-143 is a member of miR family existing in chromosome 5, which is abundant in vascular smooth muscle [13]. Data has indicated that miR-143 expression serves as an independent prognostic biomarker for colorectal cancer cells [14]. In another study, Xia et al. found that staphylococcus epidermidis could modulate cutaneous inflammatory response, and staphylococcal lipoteichoic acid could inhibit Propionibacterium acne-induced inflammation both through the inducing of miR-143 [15]. It also indicated that miR-143 may be involves in the pathological processes of various skin diseases, including immune and inflammatory responses. However, the precise role of miR-143 in the development of psoriasis vulgaris is still unknown. This study aims to investigate the correlation between miR-143 expression in peripheral blood mononuclear cell (PBMCs) and disease severity of patients with psoriasis vulgaris.

## RESULTS

### Comparison of subject baseline characteristics between the case and control groups

As shown in Table 1, there were no significant differences in age, gender, body mass index (BMI) and smoking history between the case and control groups (all *P* > 0.05). The levels of IL-17 and VEGF in the case group were higher than those in the control group (both *P* < 0.05).

**Table 1:** Comparison of baseline characteristics between the case and control groups

Characteristic	Case group(n = 194)	Control group(n = 175)	*P*
Mean age (years)	39.5 ± 12.70	40.2 ± 7.58	0.411
Gender (male/female)	120/74	94/81	0.114
BMI (kg/m^2^)	23.03 ± 3.68	23.2 ± 4.2	0.363
Smoking history (case)	105	89	0.53
IL-17 (pg/mL)	16.82 ± 4.37	8.14 ± 1.79	< 0.001
VEGF (pg/mL)	332.56 ± 82.15	94.63 ± 17.38	< 0.001

Note: age was represented as mean ± standard deviation; BMI, body mass index; IL, interleukin; VEGF, vascular endothelial growth factor.

### Relative expression of miR-143 in normal skin tissues and psoriasis vulgaris lesions

**Figure 1:**
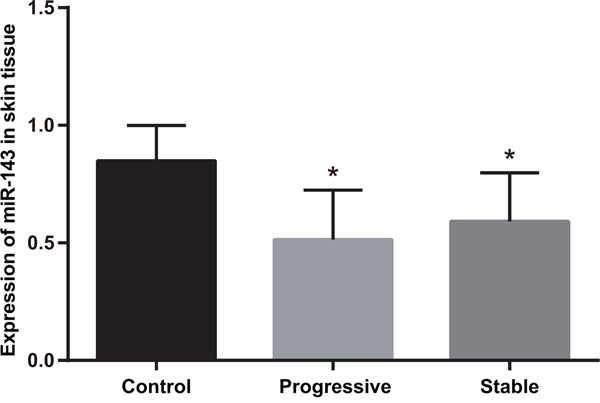
Comparison of the relative expression of miR-143 in normal skin tissues of healthy control and psoriasis vulgaris lesions of patients in progressive and stable stages Note: *, compared with the control group, *P* < 0.05; miR-143, microRNA-143.

The relative expression of miR-143 in psoriasis vulgaris lesions was significantly lower than that in normal skin tissues (*P* < 0.05) (Figure 1). Furthermore, patients in progressive stage had lower expression of miR-143 in psoriasis vulgaris lesions than those in stable stage (*P* < 0.05).

### Comparison of relative expression of miR-143 in PBMCs of subjects between the case and control groups

**Figure 2:**
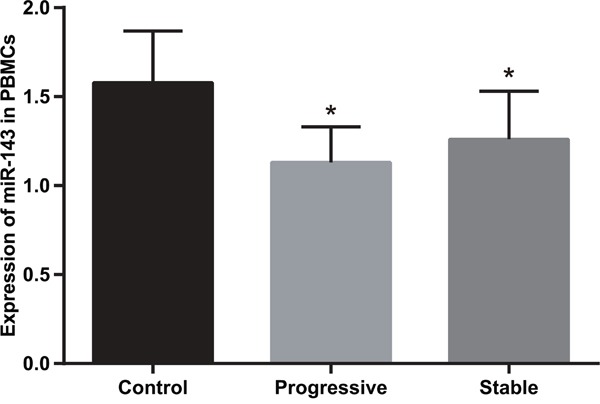
Comparison of miR-143 expression in PBMCs in subjects between the case and control groups (2^-ΔCt^) Note: *, compared with the control group, *P* < 0.05; miR-143, microRNA-143; PBMC, peripheral blood mononuclear cell.

Figure 2 indicated that the relative expression of miR-143 in PBMCs of psoriasis vulgaris patients was significantly lower than in the control group (*P* < 0.05). Similarly, patients in progressive stage also had lower miR-143 expression in PBMCs than patients in stable stage (*P* < 0.05).

### The value of miR-143 expression in PBMCs for the diagnosis of psoriasis vulgaris

**Figure 3:**
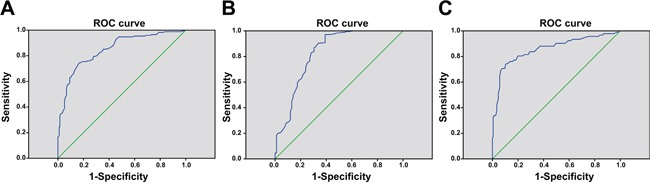
ROC curves of miR-143 expression in PBMCs for the diagnosis of psoriasis vulgaris Note: **(A)** ROC curve of miR-143 expression in PBMCs for the diagnosis of psoriasis vulgaris; **(B)** ROC curve of miR-143 expression in PBMCs for the diagnosis of psoriasis vulgaris in progressive stage; **(C)** ROC curve of miR-143 expression in PBMCs for the diagnosis of psoriasis vulgaris in stable stage; ROC, receiver operating characteristic; miR-143, microRNA-143; PBMC, peripheral blood mononuclear cell.

As presented in Figure 3A, the value of miR-143 expression for the diagnosis of psoriasis vulgaris was evaluated by ROC curve. The area under the ROC curve was 0.886 and the best cut-off value for diagnosis was 0.615. The specificity and sensitivity were 97.1% and 78.5% respectively. In addition, the specificity and sensitivity of miR-143 expression in progressive stage and stable stage of psoriasis vulgaris were evaluated by ROC curve (Figure 3B and Figure 3C). The area under the ROC curve and the best cut-off value for progressive stage of psoriasis vulgaris were 0.884 and 0.665 respectively. The specificity and sensitivity for progressive stage of psoriasis vulgaris were 90.3% and 75.5% respectively. The area under ROC curve for stable stage of psoriasis vulgaris was 0.833 and the best cut-off value was 0.615. The specificity and sensitivity for stable stage of psoriasis vulgaris were 97.1% and 60.9%, respectively. These results indicated that miR-143 was a reliable and accurate biomarker of psoriasis vulgaris.

### Correlation between miR-143 expression in PBMCs and baseline characteristics of psoriasis vulgaris patients

According to the best cut-off value of miR-143 expression in PBMCs (0.615) for diagnosis of psoriasis vulgaris, patients were divided to high-expression group (n = 84) and low-expression group (n = 90). As indicated in Table 2, the results showed that miR-143 expression in PBMCs was not significantly associated with age, gender, BMI or smoking history (all *P* > 0.05). However, miR-143 expression in PBMCs was negatively correlated with the disease course of psoriasis vulgaris (*P* < 0.001).

**Table 2:** Correlation between miR-143 relative expression and baseline characteristics of patients with psoriasis vulgaris

Characteristic	miR-143 relative expression		*P*
	High-expression (n = 98)	Low-expression (n = 96)	
Age (years)			
≤ 22	30 (30.9%)	32 (33%)	0.540
> 22	68 (69.1%)	64 (67%)	
Gender			
Male	61 (62.2%)	59 (61.5%)	0.910
Female	37 (37.8%)	37 (38.5%)	
BMI (kg/m^2^)			
18 ≤ Y ≤ 25	75 (76.5%)	70 (72.9%)	0.650
Y < 18 or Y > 25	23 (23.5%)	26 (27.1%)	
Smoking history			
Yes	50 (51.0%)	55 (57.3%)	0.381
No	48 (49.0%)	41 (42.7%)	
Disease course (year)			
≤ 3	59 (60.2%)	21 (21.9%)	< 0.001
> 3	39 (39.8%)	75 (78.1%)	

Note: miR-143, microRNA-143; BMI, body mass index.

### Correlation between miR-143 expression in PBMCs and PASI scores of psoriasis vulgaris patients

**Figure 4:**
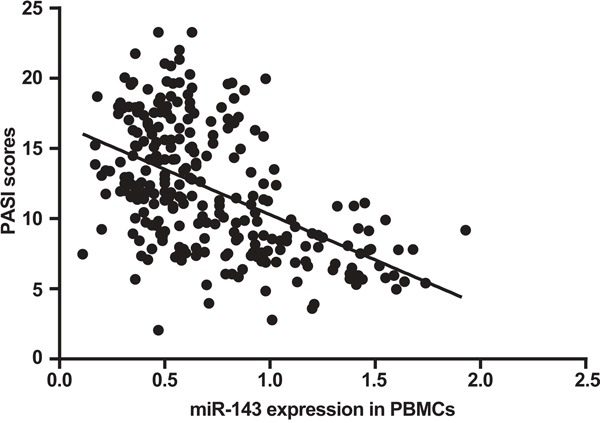
Correlation between miR-143 expression in PBMCs and PASI scores of patients with psoriasis vulgaris by Spearman rank correlation analysis Note: PASI, psoriasis area and severity index; miR-143, microRNA-143; PBMC, peripheral blood mononuclear cell.

The mean PASI scores of patients with psoriasis vulgaris were 11.22 ± 4.51. The mean PASI scores of patients in progressive stage were higher than those in stable stage (14.44 ± 3.62 vs. 7.64 ± 3.50, *P* = 0.002). The mean PASI scores of patients with high-expression of miR-143 were lower than those with low-expression of miR-143 (8.22 ± 3.20 vs. 14.27 ± 3.50, *P* < 0.05). MiR-143 expression in PBMCs was negatively correlated with PASI scores of patients with psoriasis vulgaris (r = -0.5097, *P* < 0.001) (Figure 4).

## DISCUSSION

More and more studies have attempted to gain insight into psoriasis vulgaris at a molecular level. Zhao et al. investigated the relationship between miR-210 expression in PBMCs and psoriasis vulgaris; and the results showed that increased miR-210 in PBMCs could lead to immune dysfunction by FOXP3 in CD4 (+) T cells in psoriasis vulgaris patients [16]. In another study miR-1266 showed a strong positive correlation with psoriasis vulgaris area before and after treatment and miR-1266 has potential as a blood biomarker in psoriasis vulgaris [17]. Discovery of new biomarkers would contribute to a deeper understanding of pathological mechanisms, exploring potential effective targets and providing indicators for diagnostic and prognostic tests. In this study, miR-143 expression in PBMCs of the case group was significantly lower than that of the control group, indicating that miR-143 is related to the pathological process of psoriasis vulgaris.

It is widely accepted that mature miRs are generated from long primary transcript sheared by a series of nucleases [18]. MiR-143 is one type of miRNA molecule and it has been demonstrated that Bcl-2 is a direct targeting factor for miR-143 [19]. Previous research has found that subjects with psoriasis vulgaris had lower Bcl-2 expression than healthy subjects, perhaps due to cell proliferation of cortical cells and inflammatory stimulus in psoriasis vulgaris [20]. Low expression of Bcl-2 also accounted for the shortening lifetime of cortical cells [21]. Thus, down-regulation of miR-143 would be a key factor in the pathological progress of psoriasis vulgaris.

On the other hand, our study found obvious differences in miR-143 expression in PBMCs between patients in progressive stage and patients in stable stage. Furthermore, PASI scores of patients with psoriasis vulgaris were negatively related to miR-143 expression in PBMCs. PASI score is an index of the most commonly used measurements to estimate psoriasis lesion extent in clinical practise via evaluation of erythema, infiltrate and desquamation [22]. A large number of studies have established that there were lesion-related inflammatory cytokines produced by CD8^+^ in tissues of patients with psoriasis, particularly IL-13, IL-17, IL-22 [23, 24]. These inflammatory cytokines were active in the formation of infiltrate and keratin in psoriasis, whose expression was positively correlated with PASI scores [25]. Moreover, miR-143 can suppress IL-13 expression in human mast cells [26], and so it is hypothesized that miR-143 may be involved in progression of psoriasis vulgaris via regulating IL-13 expression.

In summary, the present study provides strong evidence that miR-143 expression in PBMCs is negatively correlated with disease severity in psoriasis vulgaris and thus a low-expression of miR-143 in PBMCs would indicate poor prognosis for this disease. Thus, the targeting of miR-143 may be conducive to identifying effective therapy for psoriasis vulgaris. Unfortunately, this study only investigated the involvement of miR-143 expression in psoriasis vulgaris, and further studies are still required to fully comprehend the underlying mechanisms of psoriasis vulgaris.

## MATERIALS AND METHODS

### Ethics statement

This experiment was conducted with the approval of the Academic Ethics Committee of the First Affiliated Hospital of Zhejiang Chinese Medical University. Each patient signed written informed consent prior to study.

### Study subjects

One hundred and ninety-four patients (120 males and 74 females) diagnosed with psoriasis vulgaris from March 2014 to November 2015 in Department of Dermatology of the First Affiliated Hospital of Zhejiang Chinese Medical University and Hangzhou Hospital of Traditional Chinese Medicine were chosen by blind selection. The mean age of patients was 39.5 ± 16.70 years and the clinical course was 2 months to 7 years. Inclusion criteria were listed as follows: (1) patients with psoriasis vulgaris had typical skin lesion after explicit diagnosis [27, 28]; (2) patients had not received glucocorticoids or other drugs that can have an effect on body immunity function within previous month. Exclusion criteria were listed as follows: (1) patients who had previously received medication or ultraviolet (UVA/UVB) therapy; (2) patients who had received drug treatment for psoriasis; (3) patients with autoimmune disease, allergic disease, metabolic syndrome or other serious chronic systemic diseases. After the selection, patients were ordered into 102 cases of progressive stage (acute phase) and 92 cases of stable stage (resting phase, without inflammation development or new skin lesion) [29]. Psoriasis area and severity index (PASI) grade was assessed by the same investigator, according to the psoriasis lesion area and disease severity [30]. One hundred and ninety-four healthy subjects who underwent physical examinations in the First Affiliated Hospital of Zhejiang Chinese Medical University were selected as the control group.

### Sample collection

Five milliliters of peripheral venous blood were collected from subjects in fasting state during the early morning and then treated with the anti-coagulant ethylenediaminetetraacetic acid (EDTA). PBMCs were isolated using lymphocyte separation liquid after Ficoll density gradient centrifugation and saved at -80°C. The samples were quickly resurrected at 37°C and rinsed twice with sterile phosphate buffer solution (PBS). The cell supernatant was sucked away after centrifugation and PBMCs were prepared for further usage. Expressions of interleukin (IL)-17 and vascular endothelial growth factor (VEGF) in serum were detected in accordance with the instruction of enzyme-linked immunosorbent assay (ELISA) kit.

### Quantitative real-time polymerase chain reaction (qRT-PCR)

Total RNA was extracted from PBMCs with Trizol (Invitrogen Corporation, Carlsbad, California, USA). The concentration and purity of total RNA were measured with ultraviolet Nandodrop2000® spectrophotometer (Thermo Fisher, Waltham, Massachusetts, USA). MiRNA was reverse-transcribed with total RNA as the template and cryopreserved in -80°C. Each sample was analyzed in duplicate. PCR amplification was conducted according to instructions of PCR kits (Takara biotechnology Co., Ltd, Dalian, Liaoning, China). And 2 μL of sample cDNA was run on a Light Real Time Cycler, with the addition of 10 μL SYBR Premix Ex TaqTMII, 0.8 μL upstream primer, 0.8 μL downstream primer and 6.4 μL water. The total volume was 20 μL. U6 and miR-143 primer were synthesized by Biomics Biotechnologies Co., Ltd. (Nantong, Jiangsu, China). Cycling conditions were: pre-denaturation for 30 s at 95°C, PCR reaction with 40 cycles of 20 s at 95°C, 30 s at 60°C and 30 s at 72°C. The primer sequence of miR-143 was 5'-AGTGCGTGTCGTGGAGTC-3', (forward) and 5'-GCCTGAGATGAAGCACTGT-3' (reverse). Melting curve analysis was performed at 95°C for 5 s and 65°C for 1 s. Relative quantitative method was adopted to analyze the melting curve using U6 as an internal reference. The PCR primer sequence of U6 was 5’- GCTTCGGCAGCACATATACTAAAAT-3’ (forward) and 5’-CGCTTCACGAATTTGCGTGTCAT-3' (reverse). The fluorescent signal was observed as a single peak in the melting curve and the peak position was at the annealing temperature, indicating the credibility of PCR results.

### PASI score

The PASI scores of patients with psoriasis vulgaris were assessed by the same dermatologist. The body surface area of patients was divided into four parts, and the PASI score of each part was calculated and counted for the total PASI score. The PSI scores of patients’ body were divided as: head (10%, including neck); torso (30%, including armpits and groin); upper-limbs (20%); lower-limbs (40%, including buttocks). According the ratio of psoriasis area to body surface area, the psoriasis area of each part was divided into 7 grades: grade 0, without skin lesions (0 points); grade 1, 1%-9% (1 point); grade 2, 10%-29% (2 points); grade 3, 30%-49% (3 points); grade 4, 50%-69% (4 points); grade 5, 70%-89% (5 points); and Grade 6, 90%-100% (6 points). The grades of skin lesions were mainly evaluated by the color of erythema, the degree of infiltration and the thickness of scale. In addition, the severity of skin lesions in each part was divided into 5 grades: grade 0, without skin lesion (0 points); mild skin lesion (1 point); moderate skin lesion (2 points); severe skin lesions (3 points); extremely severe skin lesions (4 points). Total PASI score = PASI score (head) + PASI score (torso) + PASI score (upper-limb) + PASI score (lower-limb). PASI score (head) = 0.1 × (erythema + infiltration + scale) × psoriasis area; PASI score (torso) = 0.3 × (erythema + infiltration + scale) × psoriasis area; PASI score (upper-limb) = 0.2 × (erythema + infiltration + scale) × psoriasis area; PASI score (lower-limb) = 0.4 × (erythema + infiltration + scale) × psoriasis area [31].

### Statistical analysis

SPSS 21.0 software (SPSS, Inc., Chicago, Illinois, USA) was employed for statistical analysis. All measurement data were presented as mean ± standard difference (SD) and checked with normality and homogeneity of variance test. The *t* test was conducted when group mean was presented in normal distribution. Homogeneity of variance and the Mann-Whitney U test were used when group mean was not presented in normal distribution with homogeneity of variance. Count data were presented with case number, frequency or percentage and the intergroup comparison was performed using *χ^2^* test. A receiver operating characteristic (ROC) curve was carried out to evaluate the value of miR-143 expression in PBMCs for the diagnosis of psoriasis vulgaris. The skew-normal distribution of PASI scores was identified by normality test. Finally, the correlation between miR-143 expression in PBMCs and PASI scores of patients with psoriasis vulgaris was measured by Spearman rank correlation analysis. *P* < 0.05 was considered statistically significant.
